# A Functional Nexus between Photoperiod Acclimation, Torpor Expression and Somatic Fatty Acid Composition in a Heterothermic Mammal

**DOI:** 10.1371/journal.pone.0063803

**Published:** 2013-05-22

**Authors:** Fritz Geiser, Martin Klingenspor, Bronwyn M. McAllan

**Affiliations:** Biologie-Zoologie, Philipps-University, Marburg, Germany; University of Regina, Canada

## Abstract

The seasonal changes in thermal physiology and torpor expression of many heterothermic mammals are controlled by photoperiod. As function at low body temperatures during torpor requires changes of tissue lipid composition, we tested for the first time whether and how fatty acids are affected by photoperiod acclimation in hamsters, *Phodopus sungorus*, a strongly photoperiodic species. We also examined changes in fatty acid composition in relation to those in morphology and thermal biology. Hamsters in short photoperiod had smaller reproductive organs and most had a reduced body mass in comparison to those in long photoperiod. Pelage colour of hamsters under short photoperiod was almost white while that of long photoperiod hamsters was grey-brown and black. Short photoperiod acclimation resulted in regular (28% of days) torpor use, whereas all hamsters in long photoperiod remained normothermic. The composition of total fatty acids differed between acclimation groups for brown adipose tissue (5 of 8 fatty acids), heart muscle (4 of 7 fatty acids) and leg muscle (3 of 11 fatty acids). Importantly, 54% of all fatty acids detected were correlated (r^2^ = 0.60 to 0.87) with the minimum surface temperature of individuals, but the responses of tissues differed. While some of the compositional changes of fatty acids were consistent with a ‘homeoviscous’ response, this was not the case for all, including the sums of saturated and unsaturated fatty acids, which did not differ between acclimation groups. Our data identify a possible nexus between photoperiod acclimation, morphology, reproductive biology, thermal biology and fatty acid composition. They suggest that some of the changes in thermal physiology are linked to the composition of tissue and organ fatty acids.

## Introduction

Although some textbooks still describe mammals as generically ‘homeothermic’ endotherms, e.g. [1], many species belonging to more than half of mammalian orders are, in fact, ‘heterothermic’ endotherms and use torpor for energy conservation [2]–[5]. Torpor is characterized by controlled reductions of metabolic rate, body temperature (T_b_) and other physiological functions [6]. Usually, torpor is used more extensively in winter than in summer, and often the seasonal changes in torpor occurrence are controlled by photoperiod [7].

Seasonal expression of torpor is also affected by essential polyunsaturated dietary fatty acids, which have been shown to enhance the occurrence, duration and depth of torpor [8]–[15]. Data from these studies suggest that the observed diet-induced changes in the composition of tissue and cellular membrane fatty acids, which either occurred alongside, or caused the changes in thermal physiology, were to a large extent due to dietary uptake. In contrast, other recent studies have shown that seasonal changes in somatic fatty acid composition of heterothermic mammals also can occur independently of the dietary intake of fats, and may either be promoted by an endogenous circannual rhythm [16] or photoperiod acclimation [17]. Nevertheless, the available evidence on compositional changes of tissue fatty acids induced by photoperiod acclimation is essentially restricted to leg muscle of deer mice (*Peromyscus maniculatus*), which, like other members of the genus, are not strongly seasonal in their use of torpor [18]–[20].

Djungarian, or Siberian hamsters, *Phodopus sungorus,* show extreme seasonal phenotypes. During summer, they have dark brown fur, are reproductively active, with a high body mass and do not enter torpor. In autumn, they undergo a change in morphology and physiology such that by winter they have almost completely white fur, are reproductively quiescent, have a lower body mass and food intake and frequently use spontaneous (when food is available) daily torpor [7], [21]–[24]. The seasonal change in the occurrence of torpor in *P. sungorus* in autumn is controlled predominantly by the shortening photoperiod, but it can be modified somewhat by environmental temperature and food quantity and quality [7], [10], [25], [26].

Because both morphological and functional traits are so strongly influenced by photoperiodic change in *P. sungorus*, we tested the hypothesis that, even without dietary manipulations, changes in torpor use and depth induced by photoperiod acclimation are accompanied by changes of fatty acids composition of brown adipose tissue (BAT), heart, and leg muscle. BAT was investigated, because it plays an important role in non-shivering thermogenesis [27], [28], heart muscle was investigated because the heart is the most active organ during torpor [29], and leg muscle was examined to provide a comparison to previous work on deer mice [17].

## Materials and Methods

Sixteen adult *P. sungorus*, born in summer of the previous year, were held at an ambient temperature (T_a_) of 23±1°C, and a photoperiod that was adjusted weekly to the natural photoperiod of Marburg, Germany (50°48′ N, 8°45′ E). They were maintained on water and hamster chow (Altromin 7014) *ad libitum*. On 16 September they were divided into two groups (n = 8 each) of matched body mass and similar sex ratio. The Short Photoperiod (SP) group remained under natural photoperiod and at T_a_ 23°C. The Long Photoperiod (LP) group was exposed to a constant long summer solstice photoperiod of LD16∶8 at T_a_ 23°C. On 22 January when the natural photoperiod was short (LD 8.8∶15.2) for the SP group (the LP group remained under a LD16∶8 photoperiod), the T_a_ was reduced to 18°C for both SP and LP groups. Animals were kept under these conditions for eight days during which time torpor use was quantified as is outlined below. The study was carried out in strict accordance with German Animal Welfare Legislation under the umbrella of the Research Centre SFB#305, which had permission to conduct the kind of work reported here. A specific permit for this project was not required because animals were held with food *ad libitum* under mild thermal conditions and different photoperiods and then humanely sacrificed. There were no other experimental manipulations.

Throughout the experimental period hamsters were fed on Altromin hamster chow 7014, which contained 22.5% protein, 4.7% fat, 4.5% fiber, 39% carbohydrates, 11% water, minerals and vitamins. The fatty acid composition of fat is provided in Table 1 (from Altromin Tier-Labor-Service, Lage). Animals were weighed to the nearest 0.1 g with an electronic balance. The pelage index was determined by a staging system using fur coloration following [21]. The extreme values in this staging system are “1” for brown summer animals and “6” for white winter animals. Torpor was quantified between 0930 and 1030 hours by measuring body surface temperatures (T_s_) using an infrared radiation thermometer (Heiman KT 17; accuracy ±0.2°C at a distance of 1 to 40 cm; measured area 16–18 mm diameter). For each T_s_ measurement, conducted on four of the eight days the hamster were exposed to T_a_ 18°C, the back and head surface of each animals was scanned from a 2–5 cm distance and the maximum T_s_ was recorded [23]. Animals with a T_s_ <25.0°C at T_a_ 18°C were considered torpid because these T_s_s correspond with T_b_s of <31°C [10].

**Table 1:** Percent fatty acid composition of hamster chow.

Fatty acid	Dietary fat %
14∶0	0.3
16∶0	12.7
16∶1	0.3
18∶0	3.6
18∶1	21.8
18∶2n6	49.6
18∶3n3	6.8
20∶0	0.4
20∶1	0.9
20∶4n6	1.2
20∶5n6	0.5
Rest	1.9

The left column shows the numbers of carbons in the chain before the colon, followed by the number of double bonds after the colon and, for n3 and n6 polyunsaturated fatty acids, the position of the first double bond in the chain with respect to the terminal methyl group.

On 31 January, after animals had been at T_a_ 18°C for 8 days, they were humanely sacrificed with carbon dioxide and dissected for removal of inter-scapular BAT, hearts, and upper leg muscle (biceps femoralis) of n = 4 males from each group. White adipose tissue (WAT) was not examined because it showed limited response to photoperiod acclimation in *P. maniculatus* [17]. Tissues were removed and immediately frozen at −20°C for fatty acid analyses. Ovary and uterus, paired testes, epididymis and hearts were also removed and weighed to the nearest 0.01 g.

Fatty acid analyses were conducted within one week of tissue preparation. Total fatty acids were extracted from homogenized tissues and analyzed by gas chromatography as described [10]. Each sample was run twice and the mean values for fatty acids present at >0.1% are reported in the Tables. Unidentified fatty acids amounted to <2% of those identified.

Data of groups did not differ significantly between sexes for occurrence of torpor and pelage scoring (see Table 1). Therefore data were pooled and occurrence of torpor and pelage index between experimental groups were tested for differences using a Kruskal-Wallis test. General linear models were used to examine T_s_, heart mass and body mass. T-tests were used to compare means of masses of reproductive organs between treatment groups. Fatty acid percentage values were arcsine-transformed before testing means for differences by t-tests on normally distributed data (Anderson-Darling test). Bonferroni adjustments were not made because they substantially reduce the power of rejecting an incorrect null hypothesis in each test. To examine possible correlations between thermal biology (i.e. T_s_) and fatty acid composition, linear regressions were fitted using the least squares method. Numeric values are expressed as means ±1 SD; ‘n’ is the number of individuals, ‘N’ the number of measurements.

## Results

### Morphology and Torpor Use

Body mass changed with season and photoperiod acclimation (group p<0.01; Group × Initial vs Final p<0.01; Table 1). Body mass change was also significantly different between sexes, with males generally heavier than females at the end of the experiments, although body masses did not differ at the beginning of the experiments (Sex, p = 0.02; Sex × Initial vs Final p<0.05; Table 1). Heart mass (0.19±0.01 g) did not differ between sexes or treatment groups.

Mean mass of paired testes differed substantially (p = 0.001) between the SP (0.24±0.22 g) and LP (0.95±0.10 g) groups (Table 1). The mean epididymis mass in the SP group was only 0.06±0.02 g, but was about 4-times heavier (0.25±0.06 g) in the LP group. Similar to the reproductive organs in males, the combined uterus and ovary mass in females was only 0.06±0.02 g in the SP group and was heavier under LP at 0.21±0.03 g.

The pelage index was 1 (brown) for all individuals of both groups of hamsters in September. In January it remained at 1 in the LP group, but had increased significantly to a mean of 5.1 (largely white) in the SP group (Table 1).

**Figure 1:**
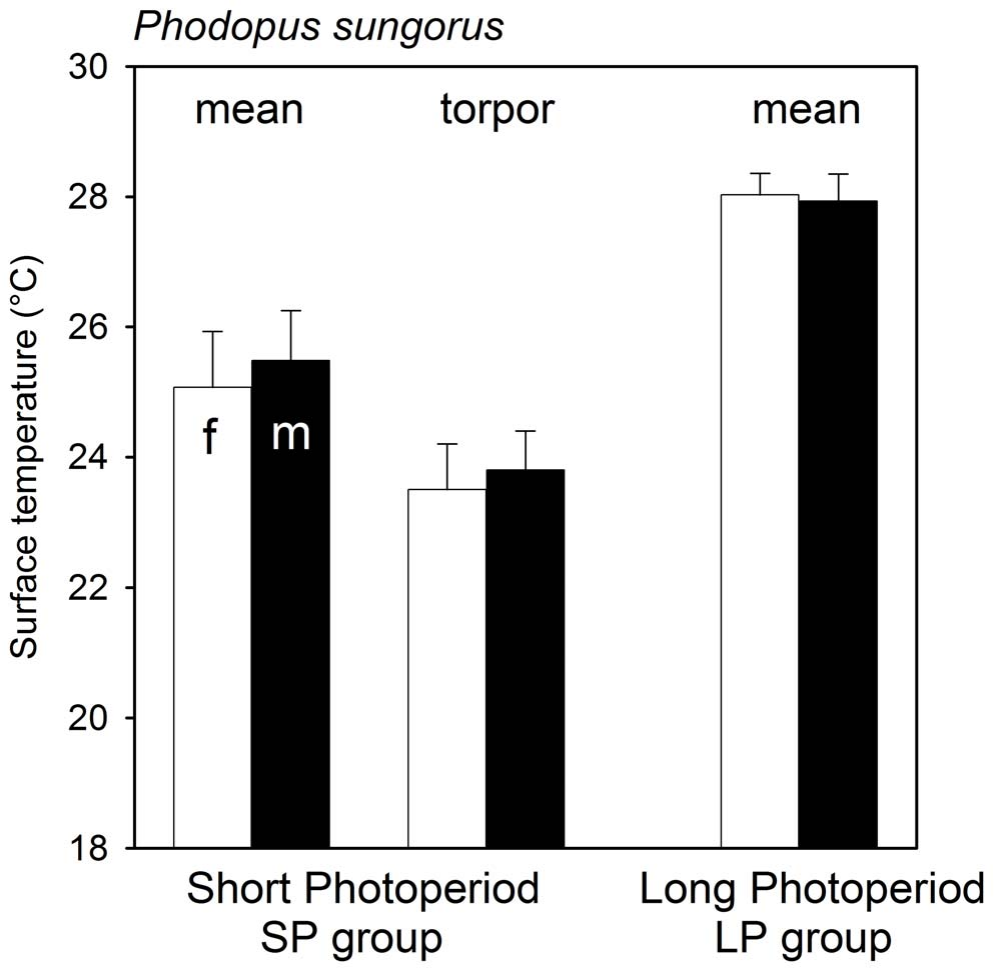
Mean surface temperatures (±1SD) female (f) and male (m) *Phodopus sungorus* acclimated to short and long photoperiods and maintained at T_a_ 18°C. Values for torpid individuals are shown only for the short photoperiod group because none of the long photoperiod animals displayed torpor.

Torpor use at T_a_ 18°C differed significantly (H_1_ = 8.52, p<0.01) between LP (0% occurrence N = 32) and SP (28.1±20.9% occurrence, N = 32) groups (Table 2). Torpor use did not differ between sexes. Similarly, the mean of all T_s_ measured in the SP group (males 25.5±0.8°C; females 25.1±0.9°C) was significantly lower (F_1,15_ = 72.3, p<0.001) than that for the LP groups (males 27.9±0.4°C; females 28.0±0.3°C), but did not differ between sexes (F_1,15_ = 0.24, p = 0.63). The mean T_s_ in torpid individuals exclusively, which only was observed in the SP group (Fig. 1), was almost identical in females and males (females T_s_ 23.5±0.7°C; males T_s_ 23.8±0.6°C).

**Table 2:** Morphological variables, pelage index and torpor occurrence of *Phodopus sungorus* acclimated to short (SP group) and long (LP group) photoperiods, number of individuals reflecting all sample sizes in parentheses.

	Body mass Initial	Body mass Final	Testes	Epididymis	Uterus+Ovary	Pelage index	Torpor occurrence (%)
	(g)	(g)	(g)	(g)	(g)		
SP females	32.1±0.7 (3)	23.4±3.6*			0.06±0.02	5.0±0.0	33.3±28.9
SP males	31.1±2.3 (5)	33.5±12.3	0.24±0.22	0.06±0.02		5.2±0.5	25.0±17.7
LP females	30.1±1.6 (4)	36.0±6.6			0.21±0.03	1.0±0	0±0
LP males	32.8±3.9 (4)	46.3±5.4*	0.95±0.10	0.25±0.06		1.0±0	0±0
Statistics	group F_ 1,31_ = 7.83 p<0.01		T_7_ = 6.49	T_6_ = 5.92	T_5_ = 8.16	H_1_ = 14.22	H_1_ = 8.52
	Sex F_1,31_ = 6.19 p = 0.02		p = 0.001	p = 0.01	p = 0.001	p<0.0001	p = 0.004
	Initial vs Final F_1,31_ = 2.21 p = 0.15						
	Group × Sex F_1,31_ = 0.18 p = 0.66						
	Group × Initial vs Final F_1,31_ = 8.34 p<0.01						
	Sex × Initial vs Final F_1,31_ = 4.39 p<0.05						
	Group × Sex × Initial vs Final F_1,31_ = 0.15 p = 0.70						
	*Indicates final body mass significantly different from initial body mass at P<0.05						

Data are means ±1 standard deviation.

### Fatty Acid Composition

Eight fatty acids were present at >0.1% in BAT (Table 3). Five of these differed significantly between the SP and LP group. All fatty acids with 16 carbons or less were present in lower concentrations in the SP than LP group. In contrast, stearic acid (18∶0) and eicosenoic acid (20∶1) were present at higher concentrations in the SP than LP group. Sums of saturated (SFA), unsaturated (UFA), polyunsaturated (PUFA), and of n3 and n6 fatty acids did not differ between treatments.

**Table 3:** Percent fatty acid composition (>0.1%) of brown adipose tissue (BAT).

Fatty acid	Short Photoperiod (n = 4)	Direction of Change	Long Photoperiod (n = 4)	T-test P-value
14∶0	0.8±0.1	<	1.2±0.2	<0.05
16∶0	16.7±1.0	<	21.1±1.3	<0.01
16∶1	2.4±0.9	<	5.3±1.2	<0.05
18∶0	14.8±1.5	>	9.5±1.4	<0.01
18∶1	41.4±1.9		40.7±1.3	ns
18∶2n6	21.7±0.6		21.4±1.2	ns
18∶3n3	0.8±0.1		0.9±0.1	ns
20∶1	0.6±0.1	>	0.1±0.2	<0.01
Rest	0.9±0.6		0.1±0.2	
SFA	32.3±1.2		31.7±1.3	ns
UFA	66.9±1.5		68.3±1.3	ns
PUFA	22.5±0.7		22.2±1.2	ns
n3	0.8±0.1		0.9±0.1	ns
n6	21.7±0.6		21.3±1.2	ns

In heart muscle seven fatty acids were identified at >0.1% (Table 4). Of these, four differed significantly between treatments, with palmitic acid (16∶0) and docosahexaenoic acid (22∶6) present at lower concentrations in the SP than LP group, whereas the PUFAs linoleic acid (18∶2) and arachidonic acid (20∶4) were present at higher concentrations in the SP than LP group. Moreover, the sum of n3 fatty acids, represented only by 22∶6, was lower in the SP than LP group and the opposite was the case for the sum of n6 fatty acids. Sums of saturated and unsaturated fatty acids did not differ.

**Table 4:** Percent fatty acid composition (>0.1%) of heart muscle.

Fatty acid	Short Photoperiod (n = 4)	Direction of Change	Long Photoperiod (n = 4)	T-test P-value
16∶0	17.9±0.6	<	20.4±0.9	<0.01
16∶1	0.5±0.5		0.7±0.5	ns
18∶0	21.0±2.2		19.8±1.5	ns
18∶1	18.6±2.9		17.6±0.8	ns
18∶2n6	22.3±0.9	>	20.4±1.0	<0.05
20∶4n6	5.5±1.3	>	3.2±0.2	<0.05
22∶6n3	12.7±1.5	<	16.9±1.3	<0.01
Rest	1.7±1.4		1.1±0.9	
SFA	38.9±2.2		40.1±0.7	ns
UFA	59.4±0.9		58.9±1.1	ns
PUFA	40.4±2.7		40.5±0.6	ns
n3	12.7±1.5	<	16.9±1.3	<0.01
n6	27.7±1.2	>	23.6±1.1	<0.01

Leg muscle contained eleven fatty acids at >0.1% (Table 5). Only three of these differed significantly between the SP and LP group and for these three cases (stearic acid 18∶0, arachidonic acid 20∶4, docosahexaenoic acid 22∶6) concentrations were greater in the SP than LP group. Sums of SFA, UFA, PUFA, and of n3 and n6 fatty acids did not differ between treatments.

**Table 5:** Percent fatty acid composition (>0.1%) of leg muscle.

Fatty acid	Short Photoperiod (n = 4)	Direction of Change	Long Photoperiod (n = 4)	T-test P-value
14∶0	0.6±0.4		1.1±0.1	ns
16∶0	21.6±3.2		21.6±0.6	ns
16∶1	5.2±2.3		8.1±1.5	ns
18∶0	7.3±1.5	>	4.4±0.8	<0.05
18∶1	33.9±3.3		35.9±1.3	ns
18∶2n6	23.3±7.5		23.1±1.5	ns
18∶3n3	0.8±0.2		1.0±0.2	ns
20∶1	0.4±0.4		0.1±0.3	ns
20∶4n6	2.0±0.3	>	1.0±0.3	<0.01
22∶1	0.7±0.9		0.3±0.5	ns
22∶6n3	4.1±0.4	>	2.8±0.8	<0.05
Rest	0.2±0.2		0.8±0.2	
SFA	29.5±4.1		27.1±1.2	ns
UFA	70.3±4.3		72.2±1.0	ns
PUFA	30.1±7.6		27.9±2.3	ns
n3	4.9±0.5		4.1±0.6	ns
n6	25.3±7.4		24.1±1.7	ns

## Discussion

Our study provides the first evidence that, in the absence of any alteration of dietary fats, the composition of fatty acids of BAT, heart muscle and leg muscle of *P. sungorus* changes in response to photoperiod acclimation and is correlated with the depth of torpor expressed. It shows that the seasonal change of thermal physiology and morphology in this species is largely controlled by photoperiod, in agreement with other studies, and suggests that some of the functional changes are linked to the fatty acid composition of tissues and organs.

### Morphology and Torpor Use

Changes in morphological variables such as body mass and pelage index in response to photoperiod acclimation in *P. sungorus* were pronounced. Hamsters in long summer photoperiod had a high body mass and dark fur, and acclimation to short photoperiod generally resulted in a decrease in body mass and a change in fur colour to largely white, similar to previous studies [10], [21]–[23], [30]. Interestingly, body masses of male and female hamsters in our study responded differently to short photoperiod acclimation. Whereas SP females decreased body mass as expected by about 30%, males did not show a significant change in body mass (to some extent because the data were skewed by one heavy male), although overall body mass was lower than in the LP group. The pelage index changed as predicted with SP acclimation to nearly white in both sexes.

Reproductive organs of male *P. sungorus* also changed enormously with photoperiod acclimation. Testes and also epididimys mass in the SP group decreased by ∼75% in comparison to the LP group, similar to that previously documented [31]. Few data are available detailing the adjustments in reproductive organs of females due to photoperiod acclimation in *P. sungorus* [32], but it appears that, like for males, female reproductive organs change in mass and activity. In our study, the combined uterus and ovary mass decreased by ∼70% in the SP in comparison to the LP group. It is known that in the season, in which torpor is expressed in heterothermic mammals, reproductive activity is often halted and reproductive organs regress, but this is not the case for all species [33]–[38].

Associated with the morphological changes due to acclimation to short photoperiod observed here, torpor occurrence in *P. sungorus* increased and torpor was deeper, but animals entered daily torpor exclusively like other daily heterotherms [10], [22], [23]. Interestingly however, male hamsters in the SP group showed the same response in T_s_ reduction as females (Fig. 1) despite the lack of a uniform reduction in body mass in the former, to some extent due to the skewed data from one fat male who did not enter torpor. Nevertheless, the data suggests that a reduction in body mass in autumn is not always a prerequisite for torpor expression in the species in winter, cf. [24]. Overall, occurrence of spontaneous torpor in the LP group of about 30% of days measured, as observed here, is as expected under the thermal conditions investigated [10], [22], [23]. However, this may represent an underestimate because only a single reading/day was made in the morning, which might have missed some torpor bouts that, on average, last from 3.5 to 7.4 h and commence in the early morning [23]. In contrast, it is unlikely that torpor bouts were missed in the LP group, because *P. sungorus* do not express spontaneous torpor when long day acclimated [10], [22].

### Fatty Acid Composition

The compositional changes of tissue fatty acids induced by photoperiod acclimation were substantial, but differed among tissues, as did the composition of tissues. Especially for brown adipose tissue (BAT), more than half (63%) of all detected fatty acids showed a significant change in concentration between the SP and LP group. This finding is especially interesting because in a previous similar study on white adipose tissue (WAT) in deer mice (*Peromyscus maniculatus*), only two fatty acids of those detected were found to differ between SP and LP acclimation groups [17]. This suggests that with regard to compositional changes in fatty acids to photoperiod acclimation, the response of BAT is more pronounced than that of WAT, which appears appropriate considering the important function of BAT in thermogenesis [27], [28] that go well beyond storage of energy, as is the case for WAT.

The second most pronounced compositional changes of fatty acids were observed for heart muscle, with 57% of fatty acids showing a significant change. There are few data for comparison, although seasonal changes in *M. marmota* heart phospholipids, especially for arachidonic acid (20∶4) and docosahexaenoic acid (22∶6) [16], are similar. Heart mitochondrial phospholipids also respond strongly to dietary fatty acids in hibernators [39] and *P. maniculatus* [17] and this compositional change is accompanied by pronounced changes in torpor patterns [40], as was observed here for *P. sungorus* under different photoperiods.

The least compositional changes were observed for leg muscle with only 27% of fatty acids showing significant differences between photoperiod groups. This observation is somewhat surprising, considering that *P. maniculatus* leg muscle was highly responsive to photoperiod acclimation, with 50% of its fatty acids showing significant compositional changes [17]. Perhaps the difference in the response to photoperiod acclimation reflects different extent of movement displayed by these species during torpor [41] because muscle fatty acid composition and locomotor performance are linked [42].

As in the previous study on photoperiod acclimation in *P. maniculatus* [17],, compositional changes were not observed for sums of SFA, UFA and MUFA (mono-unsaturated fatty acids) in any of the tissues investigated. However, changes for sums of n3 and n6 fatty acids were observed for heart muscle of *P. sungorus*, in agreement with data from *P. maniculatus* leg muscle. Other tissues of *P. sungorus* did not change sums of n3 or n6 fatty acids or their ratios.

**Figure 2:**
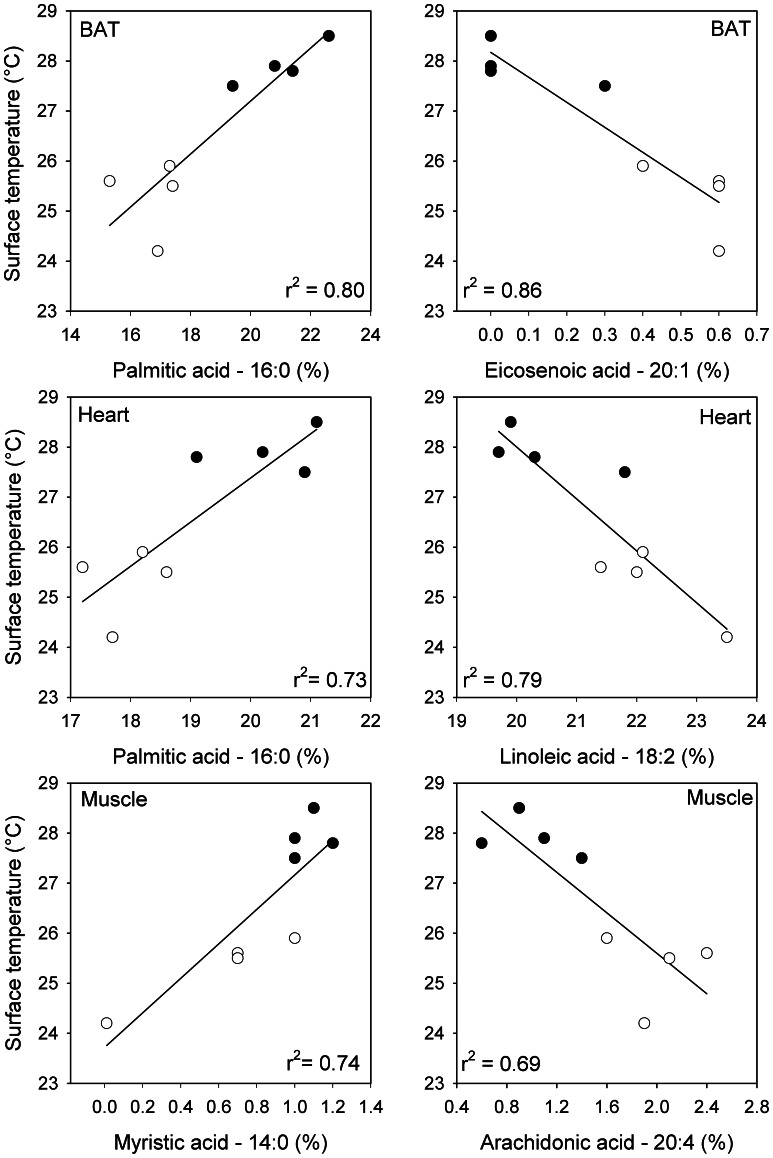
Linear regressions of mean surface temperature (T_s_) of individual *P. sungorus* as a function of fatty acid percent. Equations are provided in Table 6 and the two regressions with the greatest r^2^–values for each tissue are shown. Black dots represent long photoperiod (LP) hamsters, circles represent short photoperiod (SP) hamsters.

Changes in the amount of individual fatty acids in response to photoperiod acclimation were clearly evident, and often were highly correlated (r^2^ = 0.60 to 0.87) with the minimum T_s_ of individuals (Fig. 2; Table 6). In the absence of different dietary fatty acids these differences could be due to preferential uptake or storage, or preferential oxidation of saturated and sparing of unsaturated fatty acids during torpor [12], [43], [44], although the latter seems less likely here considering the shallow bouts of torpor expressed over a short period of time.

**Table 6:** Linear regression analyses of mean surface temperature (T_s_) as a function of fatty acid concentration.

Tissue/organ	Fatty acid	a	b	p	r^2^
BAT	14∶0	21.5	5.27	0.005	0.762
	16∶0	16.6	0.529	0.003	0.798
	16∶1	24.0	0.683*	0.009	0.709
	18∶0	31.2	−0.381*	0.017	0.64
	20∶1	28.2	−5.0	0.001	0.855
Heart	16∶0	9.95	0.871	0.007	0.733
	18∶2	48.8	−1.04	0.003	0.793
	20∶4	30.1	−0.797	0.025	0.595
	22∶6	19.6	0.475*	0.013	0.671
	n3	19.6	0.475*	0.013	0.671
	n6	41.5	−5.79	0.001	0.874
Leg muscle	14∶0	23.8	3.41	0.006	0.741(+)
	16∶1	23.1	0.532*	0.013	0.669(+)
	18∶0	30.2	0.612	0.021	0.618
	20∶4	29.6	−2.02	0.011	0.685
	22∶6	31.2	−1.33	0.018	0.635

14 fatty acids showed significant correlations with T_s_, 2 additional (+) to those that were different in t-tests. The equation is in the form of y = a+bx. Slopes that do not conform with ‘homeoviscous’ responses are indicated by asterisks (*). Examples are shown in Figure 2.

Of the five fatty acids of BAT, three changed as predicted by the ‘homeoviscous’ responses of lipids to increase in unsaturation with decreasing temperatures (14∶0 and 16∶0 were lower and 20∶1 was higher in the SP than in the LP group). In contrast, stearic acid (18∶0) and palmitoleic (16∶1) showed the opposite response, both in the comparison of means and in the regression analyses (Fig. 2; Table 6). For heart muscle, three fatty acids (16∶0, 18∶2, 20∶4) changed as predicted by a homeovicous response (Table 6), whereas docosahexaenoic acid (22∶6) was positively correlated with T_s_ and was lower in the SP than LP group, as was also observed for leg muscle of *P. maniculatus* [17]. Two of the three fatty acids showing changes to photoperiod acclimation of leg muscle went as predicted with an increase in PUFAs (20∶4 and 22∶6) in the SP group, but again stearic acid (18∶0) moved in the opposite direction in the comparison of means and palmitic acids (16∶1) also showed a positive slope as for BAT.

Astonishingly, the fatty acids 14∶0 and 16∶1, which did not show differences between groups when means were compared, were significantly correlated with T_s_ (Table 6) although only the slope for 14∶0 conforms with the concept of homeoviscosity. Thus, although pronounced compositional changes were observed for a substantial number of fatty acids, the changes induced by photoperiod acclimation are only partially explained by a homeoviscous response. It is therefore likely that interactions of specific fatty acids with membrane proteins [14], [45], [46] are responsible for appropriate adjustments that allow transient function both at low and high T_b_s during a torpor/arousal cycle. Although the observed correlation between fatty acid composition and torpor depth suggests a functional nexus, more work is needed to understand why different tissues differ in composition and the mechanisms by which tissue fatty acid composition and whole animal thermal physiology are linked.
